# Effect of lace-up ankle brace on the tibiotalar and subtalar joint during the landing

**DOI:** 10.3389/fbioe.2023.1255944

**Published:** 2023-10-12

**Authors:** Ye Luo, Mengling Hu, Zhuman Li, Xiaofan Huang, Danni Wu, Feng Li, Shaobai Wang

**Affiliations:** ^1^ School of Exercise and Health, Shanghai University of Sport, Shanghai, China; ^2^ Key Laboratory of Exercise and Health Sciences of Ministry of Education, Shanghai University of Sport, Shanghai, China

**Keywords:** dual fluoroscopic imaging system, ankle brace, tibiotalar joint, subtalar joint, *in vivo* kinematics

## Abstract

**Objective:** Ankle braces can affect the kinematics of the ankle joint during landing tasks. Previous studies were primarily relied on traditional marker-based motion capture systems, which pose limitations in non-invasively capturing the motion of the talus bone. The effect of ankle braces on the *in vivo* kinematics of the tibiotalar and subtalar joints during landing remains unknown. This study used a high-speed dual fluoroscopic imaging system (DFIS) and magnetic resonance imaging (MRI) to investigate effect of ankle braces on the *in vivo* kinematics of the tibiotalar and subtalar joints during landing.

**Methods:** Fourteen healthy participants were recruited for this study. During the experiment, static three-dimensional MRI data were collected for each participant, and 3D ankle joint models for the calcaneus, talus, and tibia were constructed. The DFIS was used to capture the images of each participant performing a single-leg landing-jump task at a height of 40 cm. The images were captured once with and without a brace in the fatigue condition, which was induced by running. The six-degree-of-freedom (6DOF) kinematic data were obtained by 2D-3D registration.

**Results:** The flexion-extension range of motion (ROM) (42.73 ± 4.76° vs. 38.74 ± 5.43°, *p* = 0.049) and anterior-posterior translation ROM (16.86 ± 1.74 mm vs. 15.03 ± 1.73 mm, *p* = 0.009) of the tibiotalar joint were decreased. The maximum inversion angle (−3.71 ± 2.25° vs. 2.11 ± 1.83°, *p* = 0.047) of the subtalar joint was decreased.

**Conclusion:** The ankle brace limited the flexion-extension ROM of the tibiotalar joints and the inversion angle of the subtalar joint during landing.

## 1 Introduction

Ankle injuries are common in athletes. Ankle sprains are one of the most frequent problems in athletes and healthcare, accounting for approximately 10%–30% of all sports injuries (Fong et al., 2007). It typically occurs in high-risk sports involving contact and frequent jumping, such as basketball, football, and volleyball (Fong et al., 2007). Ankle sprains often occur in the latter part of the season or in the second half of the game, possibly because of fatigue (Herbaut et al., 2018). Fatigue is an important factor that affects an athlete’s performance and alters lower limb biomechanics (Orishimo and Kremenic, 2006; Brazen et al., 2010). This leads to decreased dynamic stability of the lower limbs (Liu et al., 2023) and impaired proprioceptive function (Wright and Arnold, 2012), which increases the risk of ankle sprain (Gribble et al., 2004; Parijat and Lockhart, 2008).

Because of the high prevalence of ankle injuries in athletes, ankle braces are commonly used as a preventive measure to reduce the incidence of lateral ankle sprains (Olmsted et al., 2004). Ankle braces can also increase ankle stability (Mason-Mackay et al., 2016), decreasing translation and angular velocity of ankle inversion and eversion and reducing the incidence of acute ankle sprains (McGuine et al., 2011; McGuine et al., 2012). However, ankle braces may change the lower limb biomechanics during landing tasks, increasing the risk of proximal joint injuries, such as knee joint injuries (Mason-Mackay et al., 2016). Ankle braces can affect the ankle flexion-extension range of motion (ROM), which can reduce the ankle’s ability to absorb energy during jumping and landing (DiStefano et al., 2008). This leads to an increase in peak vertical ground reaction force (vGRF) during landing (Niu et al., 2016).

Previous research has mainly focused on traditional motion-capture systems (Ye et al., 2021). Traditional marker based motion-capture systems rely on tracking reflective markers adhered to the skin surface, and results can be affected by marker placement (Gorton et al., 2009) and soft tissue artifacts (STA) (Ye et al., 2021). Previous studies have mostly measured the ankle joint as a single segment (Schmitz et al., 2007; Okita et al., 2009) owing to the lack of palpable bony landmarks on the talus (Roach et al., 2021). However, this single-segment model neglects the movement between the different bones of the ankle joint (Pothrat et al., 2015; Ridder et al., 2015). It is difficult to non-invasively and accurately measure tibiotalar and subtalar joint kinematics using marker based motion-capture techniques (Ye et al., 2021). Dual fluoroscopic imaging systems (DFIS) can accurately measure bone and joint kinematics *in vivo*. To the best of our knowledge, DFIS studies on ankle bracing have investigated only the effects of ankle bracing on gait kinematics (Cao et al., 2019). The kinematic effects of ankle braces on tibiotalar and subtalar joints during landing are unclear. Therefore, this study aimed to use DFIS and magnetic resonance imaging (MRI) to determine the effects of lace-up ankle braces on the kinematics of the tibiotalar and subtalar joints during landing under fatigued conditions. We hypothesized that the ankle brace mainly limits the ROM in the flexion-extension angles of the joint.

## 2 Materials and methods

### 2.1 Participants

Fourteen healthy male participants were recruited (age: 21.6 ± 1.3 years, height: 176.9 ± 4.1 cm, weight: 69.91 ± 5.5 kg, body mass index: 22.3 ± 1.1 kg/m^2^). The inclusion criteria were as follows: 1) dominant right foot, 2) regular sport participation (at least three times a week for more than 30 min each time), 3) no chronic ankle instability, and 4) no lower limb injuries within the past 6 months. The participants’ lower limb health status was confirmed through a combination of manual examination and MRI imaging by an orthopedist. This study was approved by the Institutional Committee (No. 102772021RT133), and informed consent was obtained from all participants.

### 2.2 Instrumentation

MRI images were obtained using a MAGNETOM Prisma 3.0 T scanner (Siemens Healthcare, Erlangen, Germany) with a 16-channel foot/ankle coil. The participants were placed in a supine position with their ankle joints in a relaxed neutral position. T1W1 three-dimensional sequences were used (resolution of 0.6 × 0.6 × 0.6 mm, a flip angle of 10°, a repetition time of 10.5 ms, and an echo time of 4.92 ms).

The high-speed DFIS system consisted of two sets of X-ray emitters and receivers (diameter: 431.8 mm). In this study, the angle included was 120° and the source-to-image distance was 130 cm. The settings were 60 kV voltage, 63 mA current, and 700 μs exposure time, with an image resolution of 1,024 × 1,024 pixels. Calibration and alignment images were captured after environmental adjustments and were used to correct the inherent distortion of the dynamic images. A force plate (Kistler 9286BA, Kistler Corporation, Winterthur, Switzerland) was synchronized with the DFIS. The size was 600 mm × 400 mm × 35 mm, and the sampling frequency was set to 1,000 Hz.

### 2.3 Fatigue processes and ankle braces

To induce fatigue, the participants were asked to wear a weighted vest (16 kg) and run for 3 km. Borg scale scores ranging from 6 (no exertion at all) to 20 (maximal exertion) were used to assess participants’ feelings of fatigue (Scherr et al., 2013). The termination criterion was completion of the 3-km run and a Borg score exceeding 17, or an inability to continue running with a Borg score exceeding 19 (Tamura et al., 2016). Fatigue was successfully induced in all participants.

A McDavid Ultra-Light 195 lace-up ankle brace (McDavid Sports/Medical Products, Woodridge, IL, United States) was used. All participants used the ankle brace according to the manufacturer’s instructions. A senior user checked each participant’s application to ensure correct use. The brace has two crossing straps and a nylon buckle strap.

### 2.4 Data collection

**FIGURE 1:**
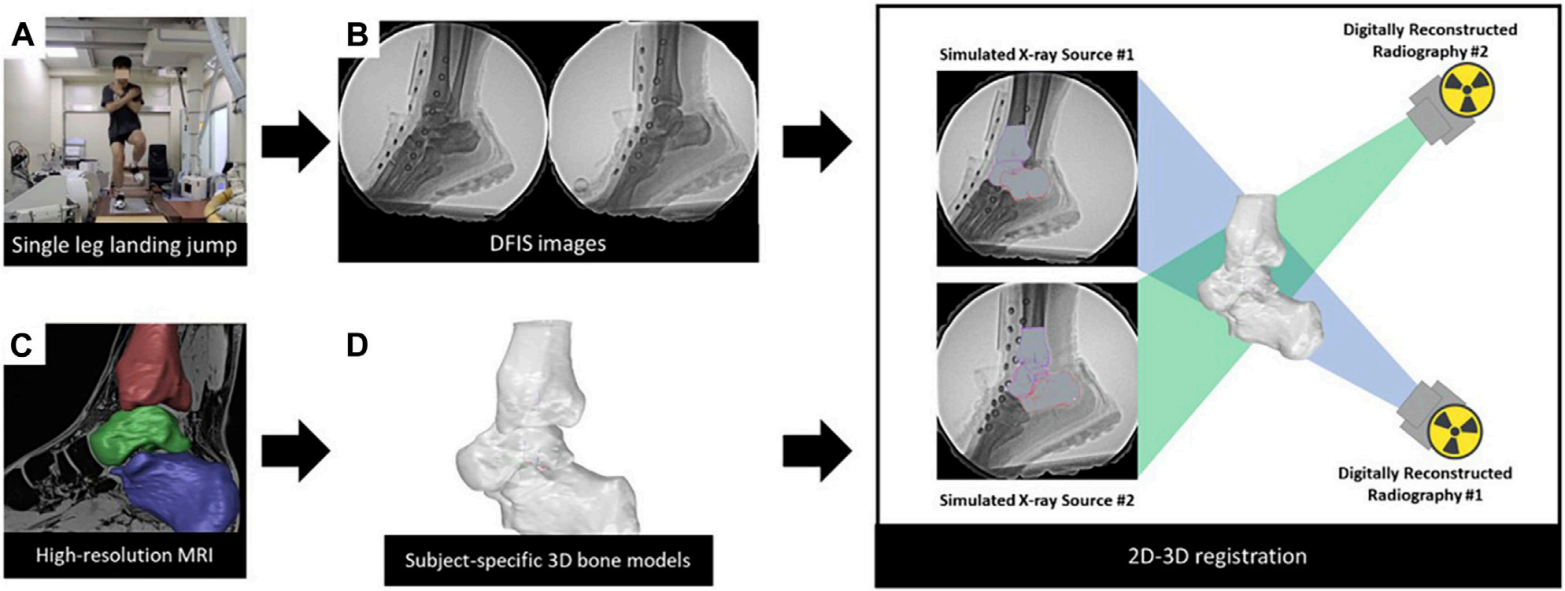
Data collection and process of the DFIS. **(A)** Participants performed a single-leg landing-jump. **(B)** Acquisition of high-speed dual fluoroscopic imaging system data. **(C)** Acquisition of individualized 3D models. **(D)** Creation of coordinate systems for each model (2D-3D registration) Combining DFIS and 3D models in a virtual environment to obtain kinematic data.

The participants were asked to wear standard clothing (vest, shorts, and shoes) and warm up before the fatigue protocol. All the participants wore traditional shoes (midsole material: ethyl vinyl acetate, thermoplastic polyurethane; heel-to-toe drop, 5 mm; upper structure, textile fabric, no arch support). Single-leg landing jumps were conducted in a random order (with/without ankle braces) after fatigue (Figure 1A). Each participant was required to perform a single-leg landing-jump on their dominant leg from a 40 cm high platform. An assistant researcher showed the test order to all participants. Several practice jumps were performed before the start of the experimental procedure to ensure the optimal performance. To minimize the radiation dose received by the participants, only one trial with and without ankle braces was conducted and analyzed (Figure 1B). The sampling frequency was set at 250 fps. The data were examined immediately after motion capture, to ensure that bones were captured in the image. The test was considered effective if only a small part of the calcaneus was out of view. A second trial was recorded only if this criterion was violated.

### 2.5 Data processing

Data processing involved the following steps: 1) Calibration of the radiographs. 2) Establishment of the hindfoot model (Figure 1C): MRI data were imported into 3D-reconstruction software (Amira 3D 2021.2, Thermo Fisher Scientific, Waltham, MA, United States ). The calcaneus, talus, and tibia were segmented manually. Segments were saved as 3D models. 3) Establishment of the coordinate system for the model (Figure 1D): A coordinate system was assigned for the 3D model, based on previous studies (Yamaguchi et al., 2009). The origin of the tibial coordinate axis was the crossing point of the longitudinal axis of the tibial shaft and tibial plateau. For the talus, the origin of the coordinate axis was at the center. The origin of the coordinate system was the midpoint of the line connecting the most lateral point of the posterior articular surface to the most medial point of the medial articular surface. This coordinate system was used to calculate the relative motion between bones. To minimize errors, this step was completed by an experienced researcher. Similar to previous studies, we use the sagittal, coronal, transverse sequence of Euler angles to describe the relative rotation between two bones (Ito et al., 2017; Negishi et al., 2022). The *x*-axis was the anterior-posterior axis, the *y*-axis was the medial-lateral axis, and the *z*-axis was the superior-inferior axis. Inversion/eversion, flexion/extension, and abduction/adduction were rotations around the x-, y-, and *z*-axes, respectively. 4) 2D-3D registration: The 3D bone model and calibrated images were imported into a 3D virtual environment simulation software (Rhinoceros 7.4, McNeel and Associates, Seattle, United States). This software was used to move each bone in 6DOF until it matched the bone contours observed on the radiographs (Figure 1). The software allowed independent rotation and translation, with increases of 0.01° and 0.01 mm, respectively. To enable data comparison between different participants, the moment of ground contact was standardized as time “0". Ground contact was defined as the instant when the force measured on the force plate exceeded 20N. Kinematics data within the first 100 ms after ground contact were processed (Myers et al., 2011). The kinematics of the tibiotalar joint refer to the motion of the talus relative to the tibia. The subtalar joint refers to the motion of the calcaneus relative to the talus. Negative values indicated lateral translation, anterior translation, superior translation, extension, inversion, and adduction of the talus relative to the tibia (calcaneus relative to the talus), whereas positive values indicated the opposite.

### 2.6 Statistics

Statistical analyses were performed using SPSS (version 27.0; IBM Corp., Armonk, NY, United States ). The kinematics and ROM of the tibiotalar and subtalar joints were calculated as means and standard deviations. A one-way analysis of variance with repeated measures was used to determine the differences in the 6DOF with and without braces. The significance level was set at *p* < 0.05.

## 3 Results

### 3.1 *In vivo* kinematics of the tibiotalar and subtalar joints

#### 3.1.1 Tibiotalar joint

**FIGURE 2:**
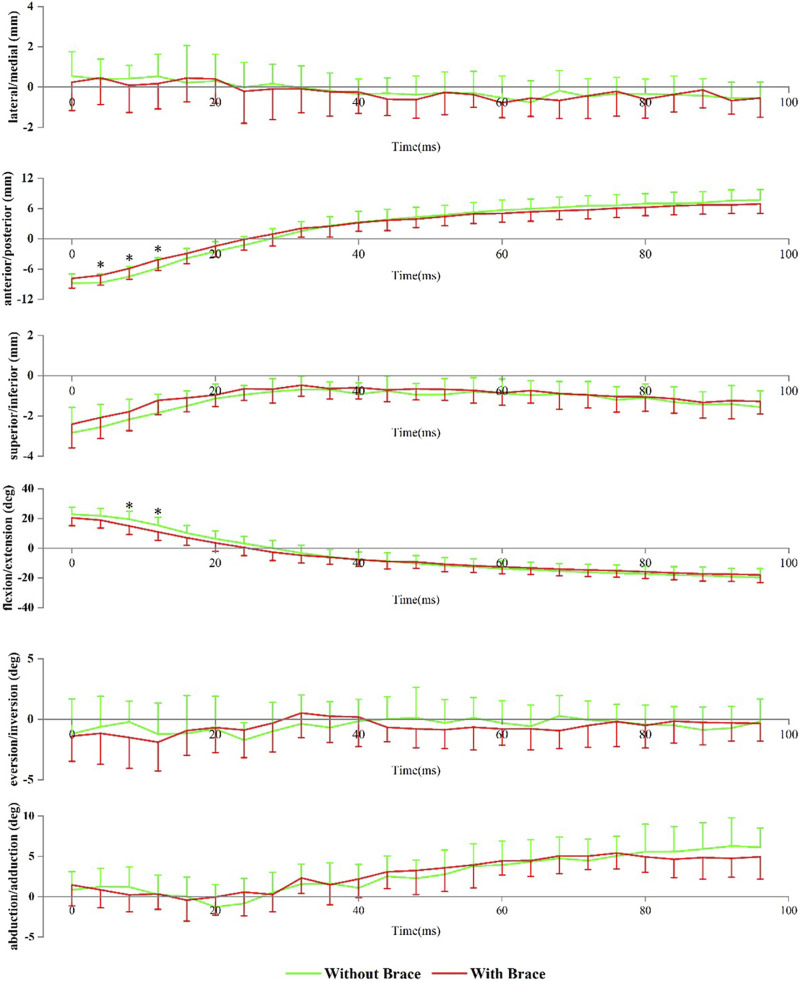
Tibiotalar joint positions during landing. Data are presented as mean values with standard deviations. Positive values indicate medial translation, posterior translation, inferior translation, flexion, eversion, and abduction, while negative values indicate the opposite. *, significant difference between with and without brace (*p* < 0.05).

Figure 2 shows the angle and translation changes of the tibiotalar joint during the landing phase in both the without- and with-brace conditions. Compared to conditions without a brace, the tibiotalar joint was more posterior at 4 ms (−8.72 ± 1.74 mm vs. −7.26 ± 1.94 mm, *p* = 0.045), 8 ms (−7.50 ± 2.00 mm vs. −5.88 ± 2.15 mm, *p* = 0.048) and 12 ms (−5.81 ± 2.01 mm vs. −4.14 ± 2.14 mm, *p* = 0.043) after the initial contact with an ankle brace. It was also more extension at 8 ms (19.44 ± 5.41° vs 14.90 ± 5.80°, *p* = 0.042) and 12 ms (15.34 ± 5.39° vs 10.88 ± 5.64°, *p* = 0.042) after the initial contact.

#### 3.1.2 Subtalar joint

**FIGURE 3:**
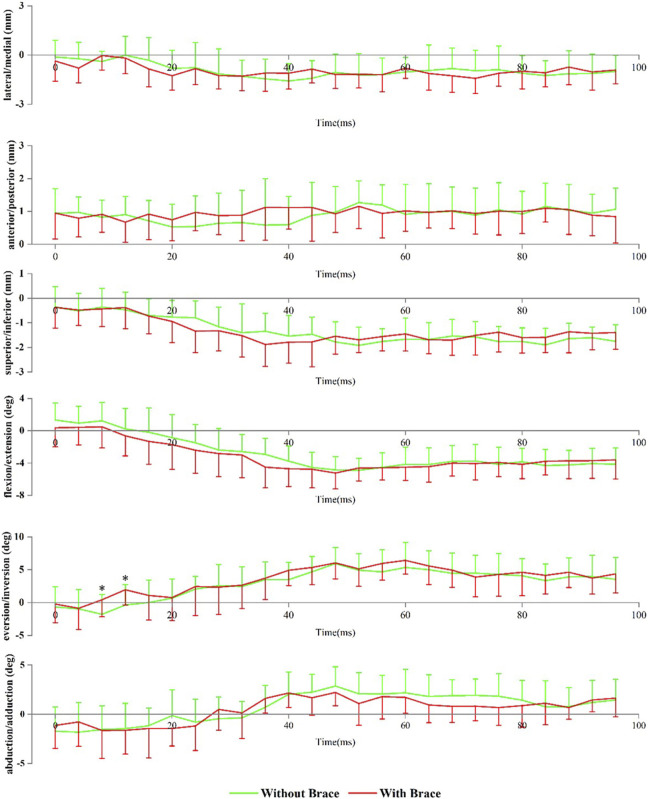
Subtalar joint positions during landing. Data are presented as mean values with standard deviations. Positive values indicate medial translation, posterior translation, inferior translation, flexion, eversion, and abduction, while negative values indicate the opposite. *, significant difference between without brace and with brace (*p* < 0.05).

Figure 3 shows the angle and translation changes of the subtalar joint during the landing phase, with and without braces. Data are presented as without a brace vs with a brace. Compared to without brace condition, wearing ankle brace conditions showed that the subtalar joint had more eversion at 8 ms (1.78 ± 3.02° vs 0.43 ± 2.60°, *p* = 0.048) and 12 ms (−0.37 ± 3.09° vs 1.96 ± 2.32°, *p* = 0.033) after the initial contact.

### 3.2 Peak translation, angles, and range of motions of the tibiotalar and subtalar joints

#### 3.2.1 Tibiotalar joint

**FIGURE 4:**
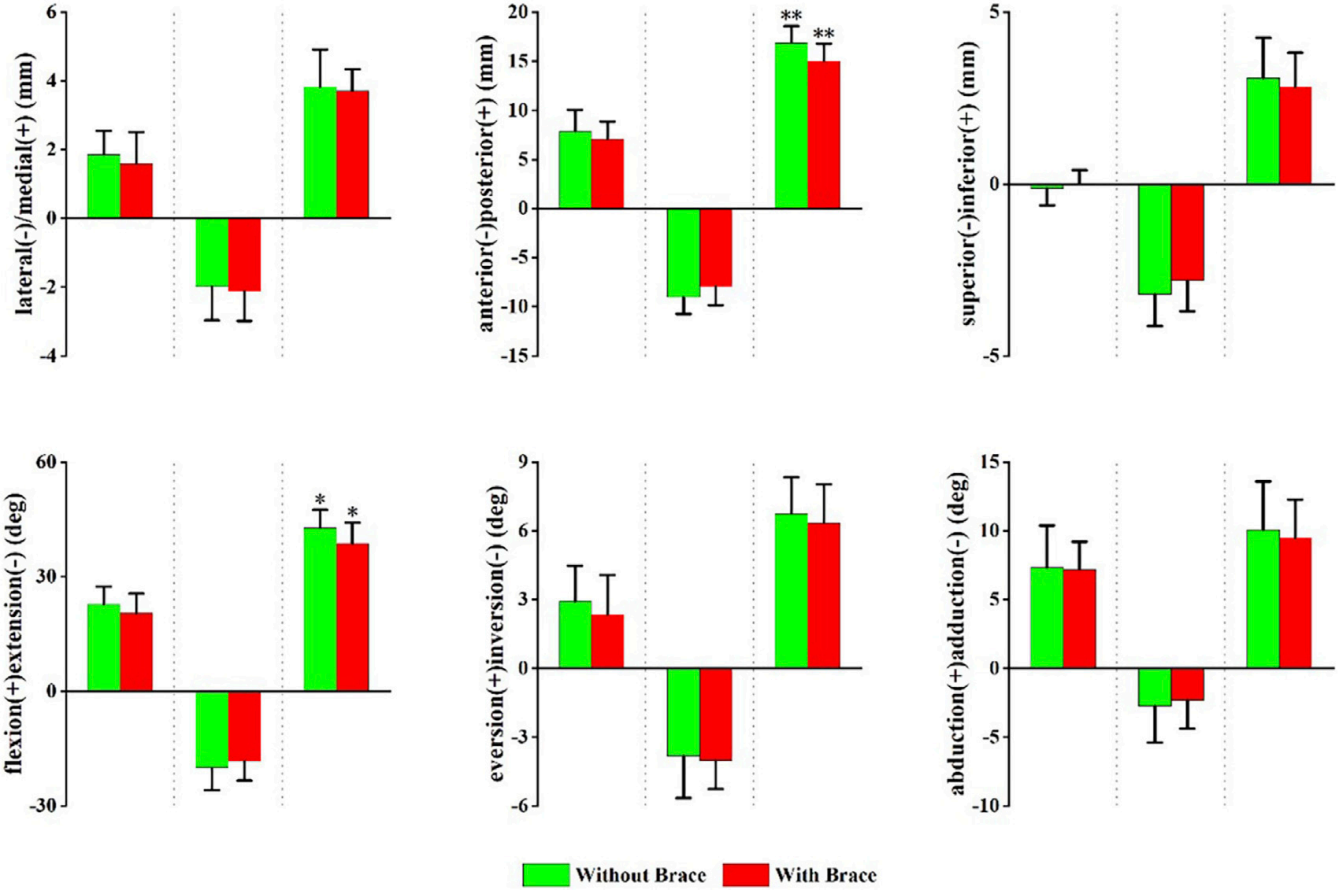
Peak translation and rotation of the tibiotalar joint with and without brace conditions. From left to right, translation and rotation directions presented in order of maximum, minimum, and range of motion (ROM). Positive values indicate medial translation, posterior translation, inferior translation, flexion, eversion, and abduction, while negative values indicate the opposite. *p < 0.05, **p < 0.01 significant difference between with and without a brace conditions.

Figure 4 shows the changes in peak translation, angle, and ROM of the tibiotalar joint when wearing ankle braces compared to those without. Data are presented as without a brace vs with a brace. Compared to without brace condition, wearing ankle brace condition resulted in a decreased flexion-extension ROM (42.73 ± 4.76° vs 38.74 ± 5.43°, *p* = 0.049) and anterior-posterior translation ROM (16.86 ± 1.74 mm vs 15.03 ± 1.73 mm, *p* = 0.009) of the tibiotalar joint.

#### 3.2.2 Subtalar joint

**FIGURE 5:**
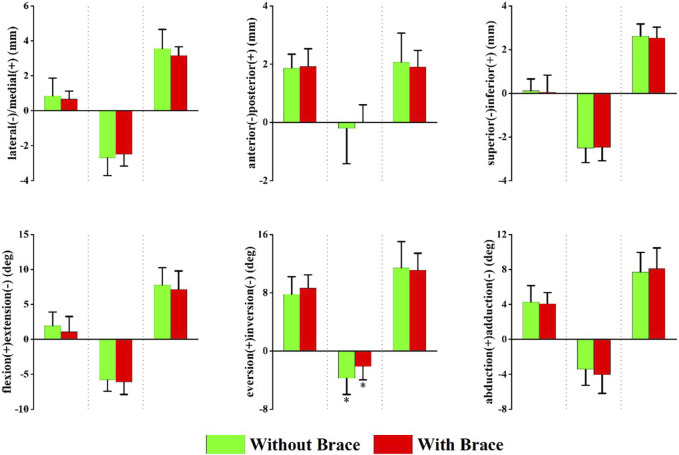
Peak translation and rotation of the subtalar joint in with and without brace conditions. From left to right, each translation and rotation direction presented in order of maximum, minimum, and range of motion (ROM). Positive values indicate medial translation, posterior translation, inferior translation, flexion, eversion, and abduction, while negative values indicate the opposite. *p < 0.05 significant difference between without brace and with brace.

Figure 5 shows the changes in the peak translation, angle, and ROM of the subtalar joint with and without ankle braces. Data are presented as without a brace vs with a brace. Compared to without a brace, the maximum ankle inversion angle decreased (−3.71 ± 2.25° vs. −2.21 ± 1.83°, *p* = 0.047) with an ankle brace.

### 3.3 Peak vertical ground reaction force

**FIGURE 6:**
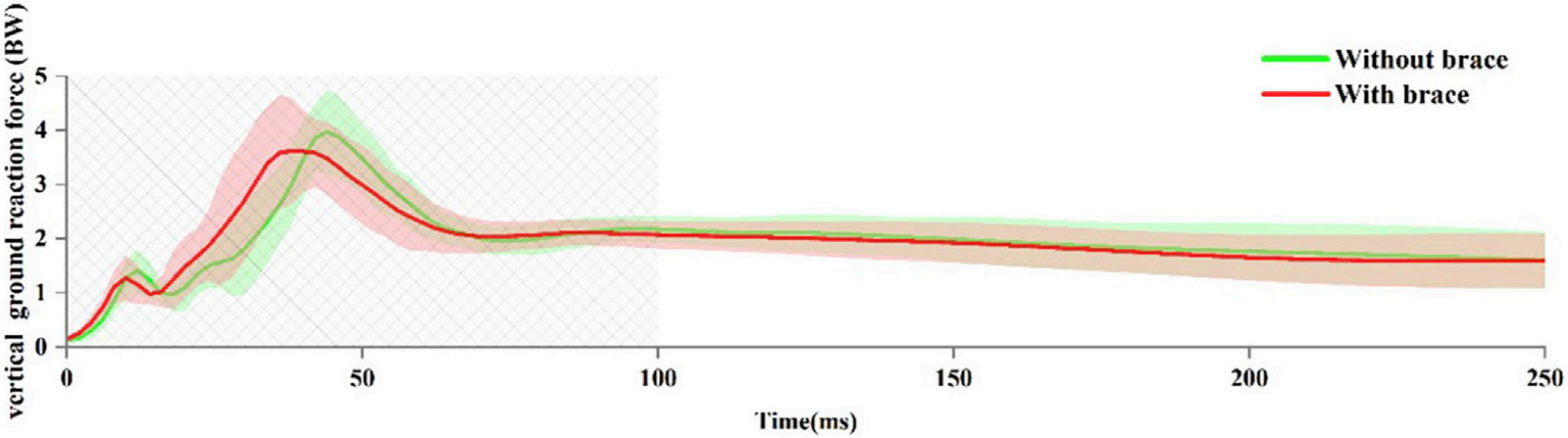
Vertical ground reaction force (vGRF) during landing. Data are presented as mean values with standard deviations. The boxed area indicates the portion of the landing phase analyzed by the high-speed dual fluoroscopic imaging system.

Figure 6 shows the changes in vGRF during Landing. Compared to without an ankle brace, wearing an ankle brace had no significant effect on peak vGRF (3.70 ± 0.60 BW vs 3.73 ± 0.59 BW, *p* = 0.664) and time to achieve peak vGRF (43 ± 5 ms vs 40 ± 7 ms, *p* = 0.2).

## 4 Discussion

This study used MRI and the DFIS to investigate the effects of a lace-up ankle brace on the kinematics of the tibiotalar and subtalar joints during landing. To the best of our knowledge, this is the first *in vivo* fluoroscopy-based study to explore this topic. Our findings showed that the brace limited tibiotalar flexion-extension ROM and reduced the maximum inversion angle of the subtalar joint. These results are partially consistent with our initial hypotheses.

Preventive ankle braces are commonly used to reduce ankle joint injuries during physical activity (McGuine et al., 2011; McGuine et al., 2012). However, the ankle brace restricts the mobility of the ankle joint complex. Most previous studies treated ankle joint as a whole, which neglects the movement between the different bones of the ankle joint (Ye et al., 2021). In these marker-based biomechanical studies, it is difficult to evaluate the specific impact of ankle braces on the tibiotalar and subtalar joints during rapid movements due to the influence of STA and footwear (DiStefano et al., 2008; Kuni et al., 2016). Our study supports and extends previous research on the effects of ankle braces on ankle joint motion (DiStefano et al., 2008). We found that wearing ankle braces limited movement in the sagittal plane of the ankle joint, mainly in the tibiotalar joint, which refers to the 6DOF between the talus and the tibia. This reduction was observed in the anterior-posterior translation and flexion-extension ROM. The reduction in flexion-extension ROM caused by ankle braces may lower the risk of ankle sprain. The mechanism of injury in lateral ankle sprains is described as a combination of inversion, flexion, and adduction (Garrick, 1977). This is because ankle stability in the maximum flexion position is significantly reduced, leading to most ankle sprains involving both inversion and flexion (Czajka et al., 2014). Therefore, the ankle brace design aims to prevent excessive frontal plane motion or to keep the ankle in a neutral position prior to landing (Eils and Rosenbaum, 2003). Our results demonstrate that the design of the lace-up ankle brace aligns with the intended design goals.

Previous studies have suggested that restricting ankle joint motion in the sagittal plane by wearing ankle braces may help prevent lateral ankle sprains. However, the sagittal plane ankle joint motion is one of the primary mechanisms for absorbing and dissipating GRF during landing (Devita and Skelly, 1992). Restricting this may result in an increase in peak vGRF during landing (Niu et al., 2016). Our study found that the ankle brace significantly restricted the ROM of flexion-extension of the tibiotalar joint. However, the vGRF and time to reach the peak vGRF were not affected by the ankle brace. This is related to the ankle brace did not significantly limit the maximum extension angle of the tibiotalar joint.

We found that wearing ankle braces decreased the maximum inversion angle of the subtalar joint during landing. This finding was consistent with that of a previous DFIS study (Zhang et al., 2019). showed that a semirigid ankle brace could significantly limit the eversion-inversion ROM of the tibiotalar and subtalar joints during walking. Reduction in the inversion angle of the subtalar joint may be an important mechanism for preventing ankle sprains (Zhang et al., 2019). In addition, the ankle brace promoted eversion of the subtalar joint before metatarsal landing (8–12 ms after the initial contact). Eversion of the subtalar joint is an important movement during weight-bearing (Czerniecki, 1988). Subtalar joint eversion increases the total contact area of the joint, which facilitates pressure distribution (Sangeorzan and Sangeorzan, 2018).

This study had the following limitations. It only examined the immediate effects of ankle braces, and the long-term effects on the tibiotalar and subtalar joints remain unknown. However, previous studies have shown that long-term ankle brace use produces effects similar to those of short-term ankle brace use (DiStefano et al., 2008). Another limitation was that only one type of ankle brace was used. The results are limited to lace-up designs with locking straps. We chose this type of brace because it is a common preventive ankle brace that is considered comfortable without rigid support. Additionally, we only included asymptomatic participants in the analysis. We have not yet analyzed individuals with common ankle conditions such as chronic ankle instability. In the future, we plan to conduct more in-depth research in this area.

## 5 Conclusion

This DFIS-based study clearly demonstrates the limiting effect of ankle braces on the tibiotalar and subtalar joints. The ankle brace limits the flexion-extension ROM of the tibiotalar joints and the inversion angle of the subtalar joint during landing. The results of this study help explain the potential mechanisms of ankle braces.

## Data Availability

The raw data supporting the conclusion of this article will be made available by the authors, without undue reservation.
